# Answer to Controversy: miR-10a Replacement Approaches Do Not Offer Protection against Chemotherapy-Induced Gonadotoxicity in Mouse Model

**DOI:** 10.3390/ijms20194958

**Published:** 2019-10-08

**Authors:** Chrysanthi Alexandri, Christina-Anna Stratopoulou, Isabelle Demeestere

**Affiliations:** 1 Research Laboratory on Human Reproduction, Université Libre de Bruxelles (ULB), 808 Route de Lennik, 1070 Brussels, Belgium; christiestrat@gmail.com (C.-A.S.); idemeest@ulb.ac.be (I.D.); 2 Obstetrics and Gynaecology, Department Mother & Child, Faculty of Medicine, University of Thessaly, Mezourlo, 41110 Larissa, Greece

**Keywords:** fertility preservation, microRNAs, apoptosis

## Abstract

It is well known that chemotherapeutic agents may lead to premature ovarian failure and infertility. Therefore, fertility preservation is highly recommended for female cancer survivors. Despite the currently available techniques, new, non-invasive methods need to be developed to protect the ovarian follicles during oncological treatments. MicroRNAs can be effective tools in this field, as they alter their expression during chemotherapy exposure, and hence they can be useful to minimize the off-target toxicity. Previously, we identified several miRNAs with an important role in newborn mouse ovaries exposed to chemotherapy; among them, the miR-10a was one of the most downregulated miRNAs. Given the controversial role of miR-10a in the ovarian function, we decided to investigate its implication in chemotherapy-induced gonadotoxicity. The downregulated levels of miR-10a were restored by a liposome system conjugated with a mimic miR-10a, and the overexpressed miR-10a prevented the upregulation of the targeted gene, phosphatase and tensin homolog (*Pten*). The apoptosis was evaluated by terminal deoxynucleotidyl transferase dUTP nick end labeling (TUNEL) Assay and *Bax* expression quantification, while histological studies were also performed to evaluate the follicle count and development. Our results showed that the miR-10a replacement could not protect the ovaries from chemotherapy-induced apoptosis, whereas the targeting of *Pten* may affect the follicle activation via the phosphoinositide 3-kinase (PI3K)/PTEN/protein kinase B (AKT) pathway. Consequently, the application of miR-10a in fertility preservation is not recommended, and the role of miR-10a needs to be further elucidated.

## 1. Introduction

Cancer is the second leading cause of death globally, and according to the latest global cancer data of the World Health Organization (WHO), one in six women develop cancer during their lifetime worldwide. However, the current advances in oncological therapies have significantly reduced the mortality rate, but still account for several undesirable side effects such as infertility. Nevertheless, thanks to improvements in cancer prognosis, increasing attention has been given to female fertility preservation. Beyond the clinically available methods to preserve fertility [1], novel, non-invasive strategies to protect the ovaries during oncological treatments are now at the pre-clinical stage, and showing promising results [2]. Alongside these experimental approaches, microRNAs have been introduced as a new potential fertoprotective strategy.

MicroRNAs or miRNAs are small, non-coding RNA molecules (21–22 nt) that can modulate genes’ expression at the post-transcriptional level [3]. MicroRNAs recognize the targeted mRNAs and bind on them by base pairing, resulting in mRNA degradation or translational inhibition [4]. The temporary modification of genes’ function has biological impact, as miRNAs regulate a wide range of cellular processes, including developmental programming, cell proliferation, and apoptosis [5]. Moreover, the role of miRNAs in ovarian function has been proven by several studies indicating their essential contribution during oogenesis, follicle activation, atresia, and in several ovarian disorders [6,7,8,9]. Given that a lot of miRNA-based therapeutics are now under clinical trials in the field of oncology [10,11] there is an increasing interest about investigating their possible application in fertility preservation strategies.

MicroRNAs present a set of attributes that make them interesting targets to be involved in the female fertility preservation concept. First of all, miRNAs have not only tissue-specific, but also cell-specific expression profiles, while one miRNA can modulate the expression of several genes, which gives the opportunity of selective and expanded targeting at the same time [12,13]. These targets may be involved in different cellular processes such as DNA damage response (DDR), apoptosis, and follicle activation, which are mainly triggered by chemotherapeutic treatments in the ovary [14,15,16]. In addition, it has been shown that miRNAs have an important role in carcinogenesis [17] and especially in tumors’ chemosensitivity**/**chemoresistance [18,19]. This means that miRNAs are themselves modulated during chemotherapy, and hence, they can be used to minimize undesirable side effects such as chemotherapy-induced gonadotoxicity.

Recently, there is accumulated interest about the role of the miR-10 family in ovarian function. The miR-10 members (miR-10a, miR-10b) consist of one of the most ancient and evolutionary conserved families among species, as they are located within the homeobox gene clusters (*Hox)* gene clusters that regulate crucial developmental processes in several animals [20,21,22]. Moreover, a lot of studies have demonstrated that miR-10 members have a role in carcinogenesis, acting as oncogenes such as miR-10b in metastatic breast cancer [23], malignant glioma [24], neurofibromatosis type 1 [25], esophageal [26] cancer, and pancreatic cancer [27]**,** while the role of miR-10a as an oncogene has been described in breast cancer [28]. However, the role of the miR-10 family in the ovary has not been elucidated yet, as controversial results have been demonstrated by different studies. According to Xiao et al., miR-10a derived from human amniotic fluid stem cells (AFSCs) has an anti-apoptotic effect on chemotherapy-damaged granulosa cells, and hence can be proposed as a future fertility preservation option [29]. On the contrary, a study of Jiajie et al. suggested that both miR-10a and miR-10b inhibit proliferation and trigger apoptosis in granulosa cells derived from human, mouse, and rat models [30]. Moreover, the possible involvement of miR-10a in human ovarian cell proliferation and apoptosis has been studied by Sirotkin and colleagues [31]. Based on this study, the transfection of miR-10a led to a significant decrease in the percentage of cells expressing proliferation markers; proliferating cell nuclear antigen (PCNA), cyclin B1 (CCNB1) and to an increase in cells expressing apoptotic markers Bcl-2-associated X protein (BAX), terminal deoxynucleotidyl transferase (TDT), cysteine-aspartic acid protease-3 (CASPASE-3).

Aiming to elucidate this controversy and draw conclusions about the potential protective effect of miR-10a, we investigated the role of miR-10a in the protection of newborn mouse ovaries against cyclophosphamide-induced apoptosis. Our findings indicate that miR-10a does not offer ovarian protection against chemotherapy-induced damage, and hence, we question its safety regarding the future clinical applications in fertility preservation.

## 2. Results

### 2.1. MiR-10a is Significantly Downregulated in Mouse Postnatal Ovaries in Response to 4-Hydroxyperoxycyclophosphamide (4-HC)

**Figure 1:**
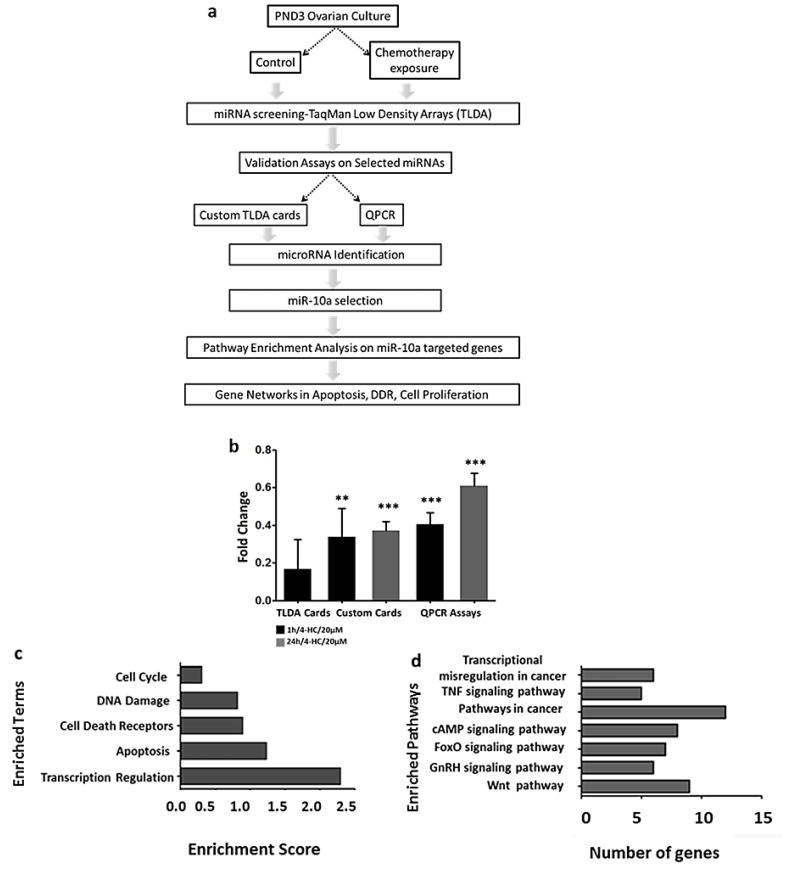
Study’s workflow for Identification of the miRNAs with ovarian protective potential**.** (**a**) Diagrammatic representation of study’s workflow and findings. The miRNA profiling was performed in PND3 ovaries cultured in vitro under control (culture medium) and chemotherapy exposure (4-HC/20μΜ). (**b**) TLDA arrays revealed that miR-10a is differently expressed after 1 h of chemotherapy exposure (*n* = 3 pairs), and the two-steps validation assays further confirmed that miR-10a was significantly downregulated after 1 h and 24 h of exposure. Validation included custom cards (*n* = 10 pairs/1 h, *n* = 6 pairs/24 h) and QPCR assays (*n* = 13 pairs/1 h, *n* = 15 pairs/24 h). The fold change is expressed as the geometric mean. The error bars correspond to standard error. ** *p*-value < 0.005, *** *p* < 0.001 by paired t-test. (**c**) Pathway enrichment analysis on the DAVID database validated by miRTarBase and (**d**) predicted targets by the TargetScan of miR-10a. MiR-10a target genes that have a key role in DNA damage response, apoptosis pathways, and cell proliferation.

In our previous study, we showed that miRNAs can alter their expression profile due to chemotherapy exposure, and this change can be observed after short exposure during 1 h at 20 µM (1 h/4-HC/20 µM) or long exposure during 24 h (24 h/4-HC/20 µM) (Figure 1a) [32]. Amongst the most affected miRNAs, which changed their expression levels after 1 h of exposure to 4-HC in mouse postnatal-day 3 (PND3) ovaries, the miR-10a was one of the most downregulated. The downregulated profile of miR-10a was validated by Custom TaqMan Low Density Arrays (TLDA) Cards and individual TaqMan QPCR Assays after 1 and 24 h of exposure to 4-HC**.** However, after 24 h, the expression was less downregulated compared to 1 h of exposure (Figure 1b).

### 2.2. MiR-10a Can Regulate a Big Network of Genes Involved in DNA Damage Response, Apoptosis, and Follicle Activation

In order to identify which pathways are regulated by miR-10a, we performed pathway enrichment analysis on the DAVID database for the validated miR-10a targeted genes (miRTarBase) and predicted (TargetScan). This in silico analysis revealed that miR-10a can regulate the expression of genes involved in important cellular processes such as cancer-related pathways, DNA damage response, apoptosis, and cell cycle (Figure 1b,c).

### 2.3. Liposome-Based Transfection of PND3 Ovaries with miR-10 Mimic

**Figure 2:**
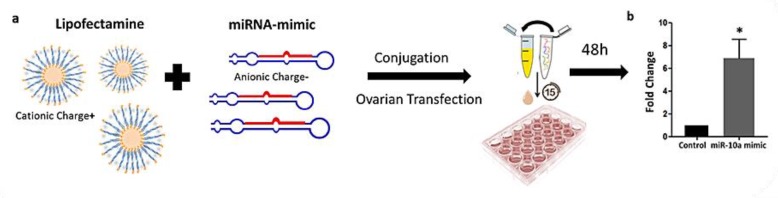
Liposome-based miR-10a mimic delivery in PND3 ovaries in vitro. (**a**) Diagrammatic representation of study’s workflow and findings. The application of miRNA replacement approaches using liposomes and miR-10a mimic molecules that could successfully increase the levels of miR-10a in PND3 ovaries in vitro. (**b**) Fold change of miR-10a expression levels of miR-10a after 48 h of transfection with miR-10a mimic using a liposome-delivery system compared to non-transfected (control) (*n* = 3). * *p*-value < 0.05. The error bars represent the standard error.

The transfection of PND3 ovaries in vitro was performed using Lipofectamine RNAiMAX, a liposome-based delivery system (Figure 2a). In our previous study, a transfection protocol was developed after testing different concentrations of the Lipofectamine and the mimic miRNA molecules, while the transfection’s efficiency was verified by a fluorescent label dsRNA [32]. After 48 h in culture with the transfection mix (Lipofectamine + Alexafluor labeled dsRNA), the fluorescent signal was detected in every ovarian section, indicating that the transfection is successful (data is not shown). In the present study, the miR-10a mimic delivery was based on the same methodology, while the levels of miR-10a were quantified by QPCR. The comparison between miR-10a mimic transfected ovaries and non-transfected ovaries (control) verified that the levels of miR-10a were significantly increased in the miR-10a mimic group (Figure 2b).

### 2.4. Mir-10a Restoration Does Not Protect the PND3 Mouse Ovaries after Exposure to 4-HC

**Figure 3:**
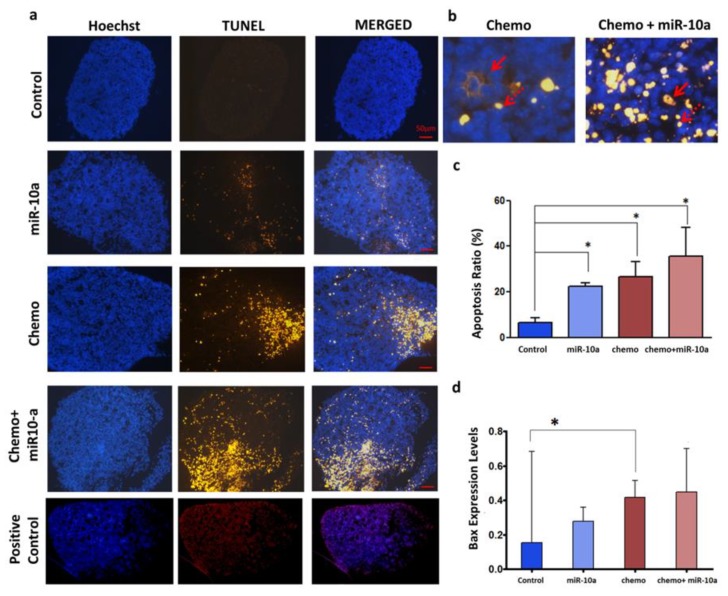
Functional analysis of miR-10a replacement approaches by apoptosis evaluation in PND3 ovaries with or without treatments. Apoptosis evaluation in PND3 ovaries with or without treatments. (**a**) Ovarian sections (5 µm) show nuclear labeling with Hoechst (blue) and apoptotic cells/TUNEL positive (yellow/red). After two days in culture, apoptosis was higher in the miR-10a mimic compared to the control group. The apoptosis level in ovaries treated with chemo (1 h/4-HC/20 µM) + miR-10a exhibit the highest rate of apoptosis compared to the control (*p* < 0.05). (**b**) Magnified merged (Hoechst/TUNEL) images of chemo and chemo + miR-10a groups indicate apoptotic oocytes (red arrow) and apoptotic granulosa cells (dashed arrow). (**c**) Quantification of apoptotic cells. The TUNEL-positive cells were quantified and expressed as a percentage of the total cells stained with Hoechst. The results are displayed as the mean of individual measurements (*n* ≥3). (**d**) The expression levels of *Bax* were quantified in the four groups. There is a trend of increasing *Bax* expression in treated groups (miR-10a, chemo, chemo + miR-10a), but the expression is significantly upregulated in the chemo group compared to the control (*n* = 7). The error bars represent the standard error, paired t-test, *p < 0.05.

To explore the possible protective effect of miR-10a in PND3 ovaries, apoptosis was evaluated in the four different groups: control, chemotherapy alone (1 h/4-HC/20 µM), miR-10a mimic alone, chemotherapy + miR-10a mimic (1 h/4-HC/20 µM/+miR-10a mimic). After 2 days, the TUNEL assay confirmed that apoptosis levels were not decreased after transfection with the miR-10a mimic in both the miR-10a mimic alone and the chemotherapy +miR-10a mimic groups in comparison to the control conditions and chemotherapy-exposed groups, respectively (Figure 3a–c). Amongst the genes involved in apoptosis, the expression of *Bax* was significantly increased in the presence of chemotherapy compared to the control (*p* < 0.05) group, but miR-10a restoration could not reduce this effect in both transfected groups with or without chemotherapy (Figure 3d).

### 2.5. MiR-10a is Involved in the PI3K/PTEN Signaling Pathway

**Figure 4:**
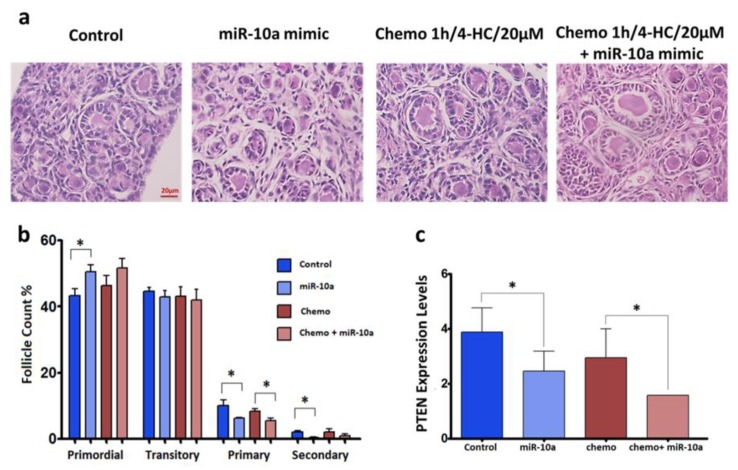
Functional analysis of miR-10a replacement approaches by follicle development evaluation. (**a**) Hematoxylin and eosin (H&E) staining on 5-µm sections of PND3 ovaries in different conditions. (**b**) The graph shows the percentage of follicles according to developmental stages (primordial, transitory, primary, secondary) in four different groups. The follicle counting was performed in at least three different experimental samples for each group (*n* ≥3). The error bars represent the standard error, *p* < 0.05. (**c**) The graph represents the expression levels of the miR-10a targeted gene, PTEN. PTEN is significantly downregulated in both the miR-10a mimic (*n* = 8) and chemo + mir-10a (*n* = 7) groups. The error bars represent the standard error, paired t-test, *p < 0.05.

In order to assess the transfection’s impact on the follicle morphology, number, and maturation stage, we performed histological studies by hematoxylin and eosin staining (Figure 3a). Primordial and growing follicles can be identified in the ovaries after 2 days in culture. The follicle counting results indicate lower numbers of primary and secondary follicles in the transfected ovaries with miR-10a compared to the other groups (Figure 4b). Moreover, the expression level of the gene *Pten* which is an experimentally validated target of miR-10a, was evaluated by QPCR. The gene was found to be significantly downregulated in both the miR-10a mimic and chemotherapy + miR-10a mimic groups compared to their corresponding controls (*p* < 0.05) (Figure 4c).

## 3. Discussion

The present study is based on the evidence that miRNAs respond to the changes of their microenvironment as they alter their expression profiles during exposure to chemotherapeutic agents. This attribute may be useful in minimizing chemotherapeutic side effects in female cancer patients such as gonadotoxicity and future infertility. MicroRNAs are tiny molecules that regulate the function of a big network of genes implicated in pathways that can be triggered by chemotherapy such as apoptosis, DNA damage response, and follicle activation. Hence, there is an increased interest to study the potential of miRNAs in reducing off-target toxicity and to identify possible targets with ovarian protective properties. An in vitro model was used in this study. The evaluation of cyclophosphamide metabolites’ toxicity on cultured mouse ovaries has revealed that 4-HC was the most gonadotoxic, because its high hydrophobicity facilitates the cellular entrance and generation of phosphoramide mustards (PM), which finally cause the damage [33]. According to the results from the study of Horicks et al., when isolated ovarian follicles were exposed to 4-HC (20 μM), both follicular survival and the oocyte maturation rate were significantly reduced [34]. As the goal of the study is to monitor the early response of miRNAs to the changes of their microenvironment and their possible implication in pathways such as apoptosis, DNA damage response, and follicle activation, the chemotherapy dosage should cause a toxic effect without destroying the follicles completely by inducing irreversible necrosis. Hence, the exposure to 4-HC for 1 h was selected as a starting point.

Using this model, we previously showed that miRNAs are modulated by chemotherapy exposure and identified potential therapeutic targets, includinglet-7a and miR-10a. According to Alexandri et al., the let-7a was able to protect the newborn mouse ovaries against chemotherapy-induced apoptosis, while the role of the let-7 family in apoptosis had also been reported in porcine granulosa cells [32,35]. In this study, we attempted to investigate the role of miR-10a in cyclophosphamide-induced ovarian damage, as it was also found to be downregulated after exposure to 4-HC in mouse PND3 ovaries. In addition, a recent study about miR-10a further confirms our findings, as cisplatin significantly reduced the expression levels of miR-10a in cancerous granulosa cells [36]. Cisplatin is an alkylating factor similar to cyclophosphamide, and we can assume that even though the mechanisms of gonadotoxicity are not exactly the same, they may induce analogous alterations on miR-10a expression profiles [37].

Proceeding to functional analysis and given that miR-10a was downregulated in mouse ovaries, we used a liposome-based delivery system to restore its function. The mimic transfer was proven to be successful, as the levels of miR-10a were significantly increased compared to control ovaries. Furthermore, the possible protective effect of miR-10 mimic transfection on PND3 ovaries was evaluated by TUNEL assay and QPCR for the selected pro-apoptotic gene, *Bax*. Regarding the TUNEL assay, it did not indicate a protective effect of miR-10a against chemotherapy-induced ovarian damage. On the contrary, miR-10a seems to induce DNA damages or apoptosis in both control and chemotherapy-exposed ovaries. Moreover, the expression of *Bax*, a key molecule in cyclophosphamide-induced apoptosis [37,38] was found to be upregulated in PND3 ovaries after 1 h of chemotherapy exposure compared to control conditions. However, its expression was not reduced after miR-10a mimic transfection. It has been shown that oocyte death induced by chemotherapeutic agents is *Bax*-dependent, as *Bax*-null mice did not display oocyte death or a loss of primordial follicles after treatment with doxorubicin. The in vivo experiments also indicate that both germ and somatic cells (granulosa cells) were protected in *Bax*-deficient female mice after chemotherapeutic treatment [39]. *Bax* is the ultimate apoptosis effector, and it has been implicated in oocyte and granulosa cell death in p53 upregulated modulator of apoptosis (*Puma)* -deficient mice [40]. In our study, we used the active metabolite of cyclophosphamide, which has been described to cause both oocyte and granulosa cell death by elevating the expression of *Bax* and triggering the mitochondrial apoptotic pathway [37]. Hence, the absence of the effect of miR-10a on *Bax* expression further suggests its inability to prevent apoptosis in both somatic and germ-derived cells after chemotherapy exposure.

In order to identify the impact of miR-10a mimic transfection on follicle activation, we performed histological studies to assess the follicle morphology and developmental stage, while the expression of *Pten*, which is an experimentally validated miR-10a target, was evaluated by QPCR [36,41,42]. The expression of *Pten* was significantly downregulated in miR-10a-transfected ovaries (*p* < 0.05) (Figure 3c). *Pten* participates in the PI3K signaling pathway, and acts as a negative regulator of phosphoinositide 3-kinase (PI3K) by transforming phosphatidylinositol-3,4,5-trisphosphate (PPI3) to phosphatidylinositol-4,5-bisphosphate (PPI2). The downregulated profiles of this gene may have an impact on the ovarian reserve, as the inhibition of *Pten* induces follicle activation. The short duration of the ovarian culture (48 h) does not favor the monitoring of notable changes regarding the follicle activation, which is a long-term process compared to DNA damages that occur shortly after chemotherapy exposure. Despite the downregulation of PTEN, the histological studies show less growing follicles (transitory, primary, and secondary) in ovaries transfected with miR-10a. This is probably due to the apoptotic action of miR-10a, which dominates the other effects that this molecule can induce regarding follicle activation. Nevertheless, the culture of the ovaries for a longer period is required to confirm the effect of miR-10a transfection on follicular activation.

Based on the evidence of our study, we conclude that miR-10a cannot protect neonatal mouse ovaries from chemotherapy-induced damage, while we showed that miR-10a upregulation induced apoptosis in healthy cultured ovaries (without chemotherapy exposure). Our results are in contrast with the study of Xiao et al. [29]**,** who claims the protective effect of miR-10a function. These controversial results harden the identification of the role of miR-10a in ovarian follicles’ apoptosis and cell proliferation. Recently, Jiajie et al. demonstrated that miR-10a can promote granulosa cells tumorigenesis through the PTEN- protein kinase B (AKT)/ wingless-related integration site (WNT) signaling by modulating the expression of *Pten*, while in a previous study, they proved that miR-10a induced follicle atresia in normal granulosa cells [36]. According to the authors, miR-10a is the “bad guy” for normal granulosa cells’ growth and development [30].

In addition, the use of miR-10a as fertoprotective therapy in cancer patients is a risky option, because several studies have demonstrated its oncogenic potential. Hence, the miR-10a replacement can interfere with oncological treatments and affect the effectiveness of the therapy. Generally, the specific miRNA delivery into targeted tissues or organs is the biggest limitation in miRNA-based therapeutics. Even if the miRNA with the ovarian protective properties is identified, an appropriate delivery system must be developed to avoid any negative implications and off-target toxicity. The delivery system should be biocompatible with low immunogenicity and toxic properties and able to transfer the miRNA into the place of action.

**Figure 5:**
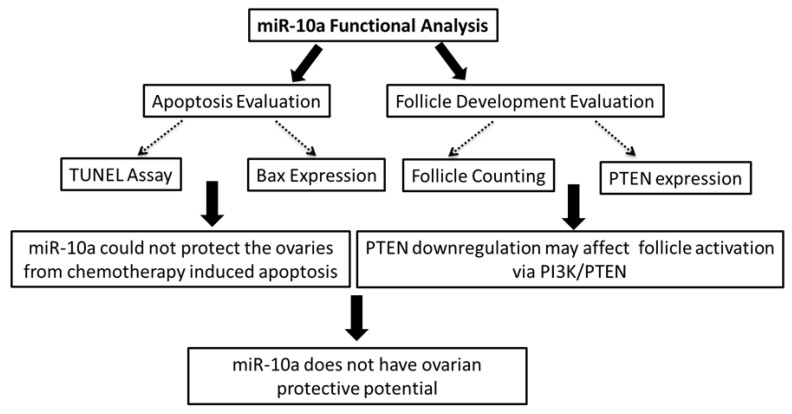
Diagrammatic representation of functional analysis workflow, findings, and conclusion. The biological effects of miR-10a restoration were evaluated by functional analysis. The apoptosis levels were evaluated by TUNEL assay and QPCR for *Bax* expression, leading to the conclusion that miR-10a was able to protect the ovaries from apoptosis. Moreover, the follicle development was also evaluated by follicle counting and QPCR for *Pten* expression. The targeting of *Pten* by miR-10a creates concerns about the implications in the PI3K/PTEN pathway and follicle activation. Consequently, miR-10a restoration did not present ovarian protective properties.

As a general conclusion, we doubt the possible use of this miR-10a as an adjuvant therapy for the protection of the ovaries during chemotherapy exposure, and we believe that more studies should be performed in order to elucidate the gene networks and pathways regulated by miR-10a (Figure 5).

## 4. Materials and Methods

### 4.1. Mice

The ovarian effects of chemotherapy exposure and miRNA replacement approaches were studied in C57BL/6xCBAF1 hybrid mice. The animals were housed in cages with controlled light (12 h light–dark cycle) and temperature conditions (22 ± 2 °C). Food and water were given ad libitum. All animal work and the experiments described in this study were approved by the local Animal Ethics Committee of “Université Libre de Bruxelles” (598N approved on 19 April 2016).

### 4.2. Culture of Mouse Ovaries in Vitro

Newborn female mice were collected on post-natal day 3 (PND3). Mice were euthanized by decapitation. The PND3 ovaries were isolated and put in Leibovitz L-15 medium (Gibco Life Technology, Merelbeke, Belgium), which was supplemented by fetal bovine serum (FBS 10%) and antibioics; 1 mg/mL of streptomycin; and 6 mg/mL of penicillin G (Sigma, Machelen, Belgium Then, the surrounding tissues were removed from the organ, and cleaned ovaries were cultured in 24-well plates. Each ovary was placed on a Millicell-CM filter membrane insert (Merck Millipore, city, Belgium) in 240 μL of Dulbecco’s Modified Eagle’s Medium (DMEM) F12 medium (Gibco Life Technology) supplemented with bovine serum albumin (BSA) (1 mg/mL) (Sigma), Albumax (1 mg/mL) (Gibco Life Technology), ascorbic acid (50 μg/mL), humane transferrin (27.5 μg/mL), penicillin G (5 U/mL), and streptomycin sulfate (3.7 U/mL) (Sigma). The culture conditions were set at 37 °C in a 5% CO_2_ humidified incubator.

The miRNA expression profiling in response to chemotherapy exposure was performed in two equal groups under different conditions. Each group contained a pool of 3–4 PND3 ovaries that have been cultured under normal (control) or chemotherapy-exposed conditions, in the presence of 4-hydroperoxycyclophosphamide (4-HC, 20 µM) (Sigma), during 1 or 24 h.

In order to evaluate the miRNA mimic transfection effects, four conditions were tested (1 ovary/condition/experiment): culture alone (control), chemotherapy exposure (1 h/4-HC/20 µM), culture with miR-10a mimic (miR-10a mimic), and chemotherapy exposure + miR-10a mimic (1 h/4-HC/20 µM + miR-10a mimic).

### 4.3. Liposome-Mediated Transfection with Synthetic miR-10a Mimic

The in vitro delivery of miR-10a mimic (300 pmol) (mirVana^®^ miRNA mimic, Life Technologies Europe BV, Merelbeke, Belgium) in PND3 ovaries in vitro was facilitated by the Lipofectamine RNAiMAX transfection reagent (20 µL) (Invitrogen, Life Technologies, Merelbeke, Belgium). The transfection mix was prepared as has been described previously [32]. The PND3 ovaries were placed on polycarbonate inserts, and they were cultured in Corning^®^ 24 Well Tissue Culture-Treated Plates using 400 µl of the transfection mix. The duration of the culture was 2 days at 37 °C in a humidified incubator with 5% CO_2_. This transfection system has been assessed and optimized in a previous study [32]. After transfection with the miRNA mimic, the ovaries were exposed to 4-HC (20 µM) or not for one hour before RNA extraction and histological evaluation.

### 4.4. In Silico Gene Expression Analysis

The prediction and identification of the miRNA targets is firstly based on the computational prediction algorithms and secondly on well-established techniques (next-generation sequencing, QRT-PCR, luciferase reporter assay, Western blot) for the target validation. There are many freely available databases for analyzing miRNAs and their targets. However, most of the miRNA targets have not been validated yet. MiRNA databases such as TargetScan (targetscan.org) include gene prediction programs to identify miRNA–mRNA interactions, while others such as MirTarBase (mirtarbase.mbc.nctu.edu.tw) provide information about the experimentally validated miRNA targets. In this study, we used information from both databases for the pathway enrichment analysis, which was performed on the DAVID database. The computational analysis showed that mir-10a is involved in pathways of DDR, apoptosis, and cell proliferation, as it targets molecules with a key role in these pathways.

### 4.5. Histological Studies and Follicle Counts

After 48 h of culture with or without treatments, the ovaries were collected and fixed in 4% paraformaldehyde for 2 h at 4 °C. Every fifth section from serially sectioned ovaries was colored with hematoxylin and eosin (H&E) staining for evaluating the follicular morphology and developmental stage: primordial, transitional, and growing (primary and secondary) were identified under light microscopy based on Gougeon’s classification [43]. Briefly, primordial follicles had a single layer of flattened cell around the oocyte; transitional follicles were identified as having a single cuboidal granulosa cell; primary follicles presented a single layer of cuboidal granulosa cells, and secondary follicles had two or more layers of granulosa cells surrounding the oocyte. Only follicles with a visible nucleus were counted.

### 4.6. TUNEL Assay

The apoptosis assessment was performed by TUNEL staining (In Situ Death Cell Detection Kit, Roche, Vilvoorde, Belgium). Briefly, ovarian sections were prepared through deparaffinization, rehydration, and were treated by proteinase K (20 μg/mL in Tris 10 mM pH 7.4, Qiagen, Venlo, Netherlands). For comparison, a positive control of induced DNA fragmentation was created by DNAse treatment. After washing steps, the sections were labeled by TUNEL agents, as it was indicated in the manufacturer’s protocol (Roche) and counterstained with Hoechst (1μg/mL). Then, the sections were observed on a Leica DM 2000 fluorescent microscope. The image analysis was performed using ZEN 2.3 software on at least three sections that were randomly selected from each ovary from three separate experiments. A lower threshold was used to reduce the background signal contribution and exclude the autofluorescence signals. The Hoechst and TUNEL-positive cells were quantified. The apoptotic level was estimated as the percentage of TUNEL-positive cells from the total Hoechst positive cells per ovary.

### 4.7. MicroRNA Extraction and Reverse Transcription

RNA enriched in miRNAs was extracted from PND3 ovaries using the ReliaPrep™ miRNA Cell and Tissue Miniprep System (Promega, Leiden, Netherlands) based on manufacturer’s instructions, and the tissue homogenization was facilitated by a pestle mixer. Moreover, the DNAse treatment step decreased the possibility of genomic DNA contamination. The measurement of RNA quantity and purity was performed on a NanoDrop spectrophotometer (Thermo Scientific, Merelbeke, Belgium).

The samples were reverse transcribed into cDNA using the TaqMan^®^ MicroRNA Reverse Transcription kit (Applied Biosystems™, Merelbeke, Belgium) and multiplexed Megaplex™ RT Primers (Applied Biosystems™, Merelbeke, Belgium) based on the manufacturer’s instructions. After reverse transcription, a pre-amplification step was followed using 2.5 µL from the cDNA product, TaqMan^®^ PreAmp kit (Applied Biosystems™) and Megaplex™ PreAmp Primers (Applied Biosystems™, Merelbeke, Belgium), as it was indicated in the manufacturer’s protocol. Then, the pre-amplified products were diluted in 100 µL of Tris-EDTA (TE) buffer (pH 8). The step of pre-amplification further increased the sensitivity of the method.

### 4.8. MicroRNA Quantification and Expression Analysis

MicroRNA expression levels were quantified by TaqMan Low-Density Arrays (TLDA), Custom TLDA Cards (as previously described by Alexandri et.al [32]), and QPCR with individual TaqMan^®^ primer probes for the following selected miRNAs: miR-10a and control genes, U6, and snoR202. Briefly, 1 µl from the diluted pre-amplified product was mixed with 1 µl of TaqMan™ MicroRNA Assays (20×) (Applied Biosystems™) and 10 µL of TaqMan™ PCR Master Mix (Applied Biosystems™) in 20 µl. All reactions were run in triplicate.

The data obtained by custom cards and individual QPCR Assays was analyzed based on the same parameters and normalization method. According to the NormFinder algorithm and DataAssist™ Software v.3.01 (Thermo Scientific, Merelbeke Belgium), U6 and snoR202 were chosen as control genes for data normalization. The data analysis was conducted in pairs of samples (control: chemo-exposed) for each single experiment. The miRNA expression level was calculated by the comparative Ct method (ΔΔCt), and the fold change (FC) was calculated by the equation 2^−ΔΔCt^. The final fold change was expressed in terms of geometric mean. The FC <0.5 corresponds to a down-regulated miRNA profile.

### 4.9. Gene Expression Quantification by QPCR

The expression of selected genes was evaluated by QPCR. Cultured PND3 ovaries that had been treated for 2 days under four different conditions were collected for mRNA expression analysis. Each collection of PND3 ovaries was subjected to total RNA extraction using the ReliaPrep™ RNA Tissue Miniprep System (Promega, Leiden, Netherlands) based on the manufacturer’s instructions. The tissue homogenization steps were facilitated using a pestle mixer. The total RNA was quantified by a NanoDrop spectrophotometer (Thermo Scientific, Merelbeke, Belgium). The total RNA and random primers were used in reverse transcription reactions.

The quantification of genes expression was performed using 2 ng of cDNA, SYBR^®^ Green Master mix (Applied Biosystems™) and 10 μM of gene-specific primers (Eurogentec, Liege, Belgium). The sequence of forward and reverse primers sequences are listed in Supplementary Table S1. Individual samples were analyzed in duplicates, and the data normalization was done by the RPL19 gene. The genes’ expression was calculated by the comparative Ct method (ΔCt), while the fold change was expressed as 2^−ΔCt^. The final fold change represents the geometric mean of the calculations.

### 4.10. Statistical Analysis

All the statistical analyses were performed on the GraphPad Prism software. For every experiment in this study, at least three biological replicates were performed, and the values are represented in terms of the mean or geometric mean ± standard error of the mean (SEM). The paired t-test was applied for data following a normal distribution, while the Mann–Whitney *U* non-parametric test was used to analyze the non-normally distributed data. A *p*-value < 0.05 was considered as a statistically significant difference.

## Supplementary Materials

The following are available online at https://www.mdpi.com/1422-0067/20/19/4958/s1.

## Abbreviations

AFSCs	Amniotic Fluid Stem Cells
PND3	Post-natal day 3
PTEN	Phosphatase and Tensin Homolog
4-HC	4-hydroxyperoxycyclophosphamide
QPCR	Quantitive Polymerase Chain Reaction
TLDA	TaqMan Low-Density Arrays
DDR	DNA Damage Response
TUNEL	Terminal deoxynucleotidyl transferase dUTP nick end labeling

## References

[1] Demeestere I., Basso O., Moffa F., Peccatori F., Poirot C., Shalom-Paz E. (2012). Fertility preservation in female cancer patients. Obstet. Gynecol..

[2] Rodriguez-Wallberg K.A., Oktay K. (2012). Options on fertility preservation in female cancer patients. Cancer Treat. Rev..

[3] Wahid F., Shehzad A., Khan T., Kim Y.Y. (2010). MicroRNAs: Synthesis, mechanism, function, and recent clinical trials. Biochim Biophys Acta - Mol. Cell Res..

[4] O’Brien J., Hayder H., Zayed Y., Peng C. (2018). Overview of MicroRNA Biogenesis, Mechanisms of Actions, and Circulation. Front. Endocrinol.

[5] Mohr A., Mott J. (2015). Overview of MicroRNA Biology. Semin. Liver Dis..

[6] Reza A.M.M.T., Choi Y.-J., Han S.G., Song H., Park C., Hong K., Kim J.H. (2019). Roles of microRNAs in mammalian reproduction: from the commitment of germ cells to peri-implantation embryos. Biol. Rev..

[7] McBride D., Carré W., Sontakke S.D., Hogg C.O., Law A., Donadeu F.X., Clinton M. (2012). Identification of miRNAs associated with the follicular–luteal transition in the ruminant ovary. Reproduction.

[8] Guo Y., Sun J., Lai D. (2017). Role of microRNAs in premature ovarian insufficiency. Reprod. Biol. Endocrinol..

[9] Toloubeydokhti T., Bukulmez O., Chegini N. (2008). Potential Regulatory Functions of MicroRNAs in the Ovary. Semin. Reprod. Med..

[10] Chakraborty C., Sharma A.R., Sharma G., Sarkar B.K., Lee S.-S., Chakraborty C. (2018). The novel strategies for next-generation cancer treatment: miRNA combined with chemotherapeutic agents for the treatment of cancer. Oncotarget.

[11] Viteri S., Rosell R. (2018). An innovative mesothelioma treatment based on miR-16 mimic loaded EGFR targeted minicells (TargomiRs). Transl. Lung Cancer Res..

[12] Penso-Dolfin L., Moxon S., Haerty W., Di Palma F. (2018). The evolutionary dynamics of microRNAs in domestic mammals. Sci. Rep..

[13] Friedman R.C., Farh K.K.-H., Burge C.B., Bartel D.P. (2009). Most mammalian mRNAs are conserved targets of microRNAs. Genome Res..

[14] Tulay P., Naja R.P., Cascales-Roman O., Doshi A., Serhal P., SenGupta S.B. (2015). Investigation of microRNA expression and DNA repair gene transcripts in human oocytes and blastocysts. J. Assist. Reprod. Genet..

[15] Zhang J., Xu Y., Liu H., Pan Z. (2019). MicroRNAs in ovarian follicular atresia and granulosa cell apoptosis. Reprod. Biol. Endocrinol..

[16] Kalich-Philosoph L., Roness H., Carmely A., Fishel-Bartal M., Ligumsky H., Paglin S., Wolf I., Kanety H., Sredni B., Meirow D. (2013). Cyclophosphamide Triggers Follicle Activation and “Burnout”; AS101 Prevents Follicle Loss and Preserves Fertility. Sci. Transl. Med..

[17] Kong Y.W., Ferland-McCollough D., Jackson T.J., Bushell M. (2012). microRNAs in cancer management. Lancet Oncol..

[18] Yu X., Chen Y., Tian R., Li J., Li H., Lv T., Yao Q. (2017). miRNA-21 enhances chemoresistance to cisplatin in epithelial ovarian cancer by negatively regulating PTEN. Oncol. Lett..

[19] Blower P.E., Chung J.-H., Verducci J.S., Lin S., Park J.-K., Dai Z., Liu C.G., Schmittgen T.D., Reinhold W.C., Croce C.M. (2008). MicroRNAs modulate the chemosensitivity of tumor cells. Mol. Cancer Ther..

[20] Tehler D., Høyland-Kroghsbo N.M., Lund A.H. (2011). The miR-10 microRNA precursor family. RNA Biol..

[21] Tanzer A., Amemiya C.T., Kim C.-B., Stadler P.F. (2005). Evolution of microRNAs located within Hox gene clusters. J. Exp. Zool Part. B Mol. Dev. Evol..

[22] Lemons D., McGinnis W. (2006). Genomic Evolution of Hox Gene Clusters. Science.

[23] Ma L., Teruya-Feldstein J., Weinberg R.A. (2007). Tumour invasion and metastasis initiated by microRNA-10b in breast cancer. Nature.

[24] Sasayama T., Nishihara M., Kondoh T., Hosoda K., Kohmura E. (2009). MicroRNA-10b is overexpressed in malignant glioma and associated with tumor invasive factors, uPAR and RhoC. Int. J. Cancer.

[25] Chai G., Liu N., Ma J., Li H., Oblinger J.L., Prahalad A.K., Gong M., Chang L.-S., Wallace M., Muir D. (2010). MicroRNA-10b regulates tumorigenesis in neurofibromatosis type 1. Cancer Sci..

[26] Tian Y., Luo A., Cai Y., Su Q., Ding F., Chen H., Liu Z. (2010). MicroRNA-10b Promotes Migration and Invasion through KLF4 in Human Esophageal Cancer Cell Lines. J. Biol. Chem..

[27] Nakata K., Ohuchida K., Mizumoto K., Kayashima T., Ikenaga N., Sakai H., Lin C., Fujita H., Otsuka T., Aishima S. (2011). MicroRNA-10b is overexpressed in pancreatic cancer, promotes its invasiveness, and correlates with a poor prognosis. Surgery.

[28] Balatti V., Oghumu S., Bottoni A., Maharry K., Cascione L., Fadda P., Parwani A., Croce C., Iwenofu O.H. (2018). MicroRNA Profiling of Salivary Duct Carcinoma Versus Her2/Neu Overexpressing Breast Carcinoma Identify miR-10a as a Putative Breast Related Oncogene. Head Neck Pathol..

[29] Xiao G.-Y., Cheng C.-C., Chiang Y.-S., Cheng W.T.-K., Liu I.-H., Wu S.-C. (2016). Exosomal miR-10a derived from amniotic fluid stem cells preserves ovarian follicles after chemotherapy. Scientific Reports.

[30] Jiajie T., Yanzhou Y., Hoi-Hung A.C., Zi-Jiang C., Wai-Yee C. (2017). Conserved miR-10 family represses proliferation and induces apoptosis in ovarian granulosa cells. Sci. Rep..

[31] Sirotkin A.V., Marcela L., Dmitriy O., Pauline B., Miloš M. (2010). Identification of MicroRNAs controlling human ovarian cell proliferation and apoptosis. J. Cell Physiol..

[32] Alexandri C., Stamatopoulos B., Rothé F., Bareche Y., Devos M., Demeestere I. (2019). MicroRNA profiling and identification of let-7a as a target to prevent chemotherapy-induced primordial follicles apoptosis in mouse ovaries. Sci. Rep..

[33] Desmeules P., Devine P.J. (2006). Characterizing the Ovotoxicity of Cyclophosphamide Metabolites on Cultured Mouse Ovaries. Toxicol. Sci..

[34] Horicks F., Van Den Steen G., Houben S., Englert Y., Demeestere I. (2015). Folliculogenesis Is Not Fully Inhibited during GnRH Analogues Treatment in Mice Challenging Their Efficiency to Preserve the Ovarian Reserve during Chemotherapy in This Model. PLoS ONE.

[35] Cao R., Wu W.J., Zhou X.L., Xiao P., Wang Y., Liu H.L. (2015). Expression and Preliminary Functional Profiling of the let-7 Family during Porcine Ovary Follicle Atresia. Mol. Cells.

[36] Tu J., Cheung H.-H., Lu G., Chen Z., Chan W.-Y. (2018). MicroRNA-10a promotes granulosa cells tumor development via PTEN-AKT/Wnt regulatory axis. Cell Death Dis..

[37] Morgan S., Anderson R.A., Gourley C., Wallace W.H., Spears N. (2012). How do chemotherapeutic agents damage the ovary?. Hum. Reprod. Update.

[38] Zhao X., Huang Y., Yu Y., Xin X. (2010). GnRH antagonist cetrorelix inhibits mitochondria-dependent apoptosis triggered by chemotherapy in granulosa cells of rats. Gynecol. Oncol..

[39] Perez G.I., Knudson C.M., Leykin L., Korsmeyer S.J., Tilly J.L. (1997). Apoptosis-associated signaling pathways are required for chemotherapy-mediated female germ cell destruction. Nat. Med..

[40] Nguyen Q.-N., Zerafa N., Liew S.H., Morgan F.H., Strasser A., Scott C.L., Findlay J.K., Hickey M., Hutt K.J. (2018). Loss of PUMA protects the ovarian reserve during DNA-damaging chemotherapy and preserves fertility. Cell Death Dis..

[41] Liu S., Sun J., Lan Q. (2013). TGF-β-induced miR10a/b expression promotes human glioma cell migration by targeting PTEN. Mol. Med. Rep..

[42] Chou C.-H., Shrestha S., Yang C.-D., Chang N.-W., Lin Y.-L., Liao K.-W., Huang W.-C., Sun T.-H., Tu S.-J., Lee W.-H. (2018). miRTarBase update 2018: a resource for experimentally validated microRNA-target interactions. Nucleic Acids Res..

[43] Gougeon A. (1996). Regulation of Ovarian Follicular Development in Primates: Facts and Hypotheses. Endocr. Rev..

