# Supplementary material for: Answer to Controversy: miR-10a Replacement Approaches Do Not Offer Protection against Chemotherapy-Induced Gonadotoxicity in Mouse Model
Source: Int J Mol Sci. 2019 Oct 8;20(19):4958. doi: 10.3390/ijms20194958 (PMC6801898; doi:10.3390/ijms20194958)
Supplement: Supplementary file 1 [file ijms-20-04958-s001.pdf]

Supplementary Table 1. Gene Primers Sequences

Gene Symbol	Official Name	Primers' Sequence (Forward-Reverse)
Pten	phosphatase and tensin homolog	F: TTC-ACG-TCC-TAC-CCC-TTT-GC<br>R: GGT-CCA-GAG-CCC-AGG-TAG-AA
Bax	BCL2-associated X protein	F: TTG-TAA-TTC-ATC-TGC-CGC-CG<br>R: AGG-GTT-TCC-AGA-TTG-GGT-CC
Rpl19	ribosomal protein L19	F: GAA-AGG-TGC-TTC-CGA-TTC-CA<br>R: TGA-TCG-CTT-GAT-GCA-AAT-CC
